# Protein folding and unfolding: proline *cis*‐*trans* isomerization at the *c* subunits of F_1_F_O_‐ATPase might open a high conductance ion channel

**DOI:** 10.1002/prot.26383

**Published:** 2022-05-19

**Authors:** Salvatore Nesci

**Affiliations:** ^1^ Department of Veterinary Medical Sciences University of Bologna via Tolara di Sopra, 40 Ozzano Emilia 40064

**Keywords:** *cis*‐*trans* isomerization, *c*‐ring, F_1_F_O_‐ATPase, mitochondria, permeability transition pore

## Abstract

The *c* subunits, which constitute the *c*‐ring apparatus of the F_1_F_O_‐ATPase, could be the main components of the mitochondrial permeability transition pore (mPTP). The well‐known modulator of the mPTP formation and opening is the cyclophilin D (CyPD), a peptidyl‐prolyl *cis*‐*trans* isomerase. On the loop, which connects the two hairpin α‐helix of *c* subunit, is present the unique proline residue (*Pro*_40_) that could be a biological target of CyPD. Indeed, the proline *cis*‐*trans* isomerization might provide the switch that interconverts the open/closed states of the pore by pulling out the *c*‐ring lipid plug.

## 1 THE *c* SUBUNITS ARCHITECTURE OF THE F_1_F_O_‐ATPASE

**FIGURE 1:**
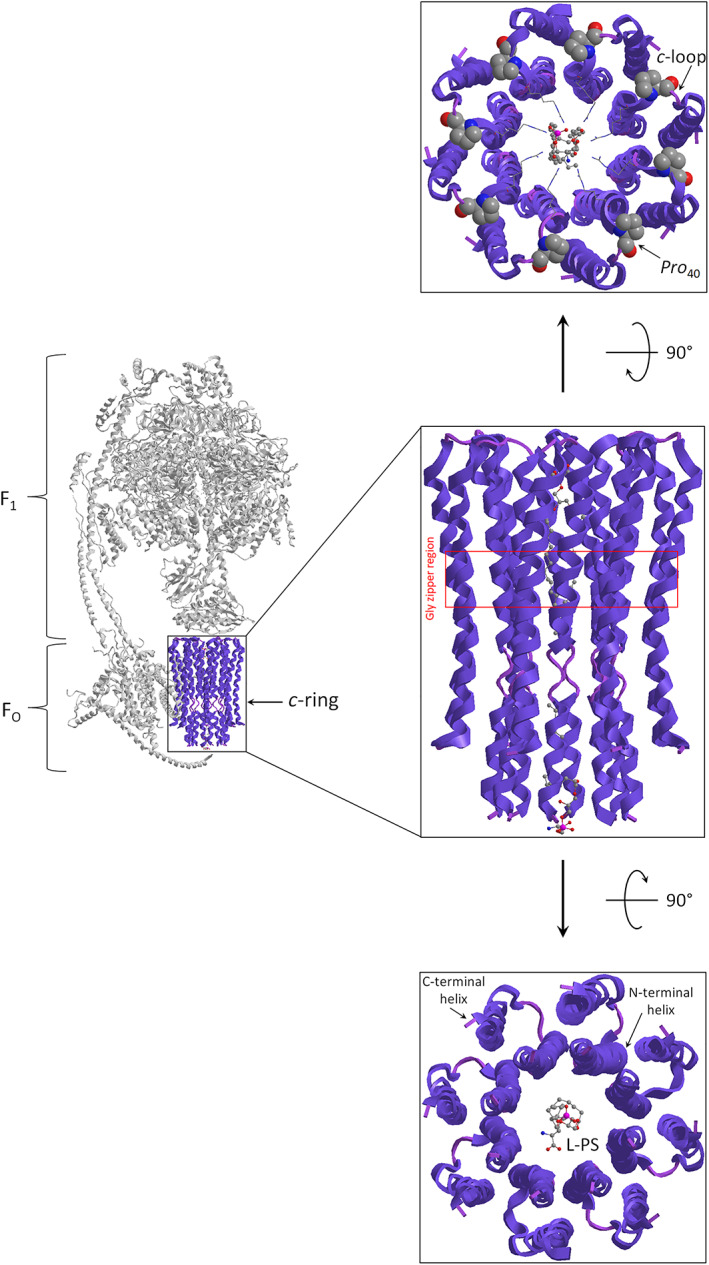
The *c*‐ring structure of F_1_F_O_‐ATPase is obtained from modified PDB ID code: 6TT7. On the right, the structure of mammalian F_1_F_O_‐ATPase with the *c*‐ring highlighted in purple. On the left, the spatial arrangement of the *c* subunits in cartoons mode. In the middle, the *c*‐ring structure is viewed laterally. The red box highlights the region of the glycine zipper present on the N‐terminal helix. On the top, the *c*‐ring is viewed from the matrix. In the hole of the *c*‐ring, the phosphatidylserine (as a ball‐and‐stick model) is specifically coordinated with the *Arg*_38_ residues drawn as a stick on the N‐terminal helix of each *c* subunit. The *Pro*_40_ is illustrated as a space‐filling model on the *c*‐loop. The *c*‐ring is viewed from the cytoplasm side of the IMM (lower panel). The lyso‐phosphatidylserine (L‐PS) as a ball‐and‐stick model is depicted in the middle of the *c*‐ring

The *c*‐subunits of F_1_F_O_‐ATPase are multifunctional proteins whose energy transduction features cover the transmembrane H^+^ translocation, whereas stoichiometry determines the species‐specific bioenergetic cost of ATP [1]. The helical hairpin structure of the *c* subunit has the N‐ and C‐terminal faced on the cytoplasm side of the inner mitochondrial membrane (IMM), whereas the amino acids that form the loop region (*c*‐loop) and connect the two transmembrane α‐helix are faced on the matrix side of IMM. Moreover, the loops of each *c* subunit are joined to the foot of the hydrophilic F_1_ portion of the enzyme. The annular arrangement of *c* subunits oligomer, which constitutes the so‐called *c*‐ring, has two concentric circles. The inner circle is composed of the N‐terminal helix, whereas the outer circle is of the C‐terminal helix of *c* subunits (Figure 1). These packed hairpins form a sort of hourglass seen laterally from the membrane side. In the middle of the membrane, the concavity of the *c*‐ring hosts on the C‐terminal helix the H^+^ binding site that is represented with a conserved *Glu*_58_ residue in the mitochondrial F_1_F_O_‐ATPase [2]. In addition to this, to ensure the rotation of the rotor driven by protonmotive force (Δ*p*) in the ATP synthesis mode or the opposite function of the ATP hydrolysis with the Δ*p* dissipation, the carboxylate side chains of *Glu*_58_ switch from deprotonated to protonate conformation during the ion translocation. Indeed, the H^+^ binding site exposed to a hydrophilic environment within the half‐channels of *a* subunit is oriented in an outward‐facing open conformation (the carboxylic group adopts the unlooked conformation). On the contrary, in the hydrophobic environment of the IMM the carboxyl group re‐orients towards an inward‐faced closed conformation (H^+^ locked conformation) during the rotor rotation with a favoured energy state to enter in the IMM [3].

Importantly, the *c*‐ring is filled with two different phospholipids at the two opposite sides of its cavity (Figure 1). At the matrix side, a phosphatidylserine (PS) is anchored by electrostatic coordination to the positive charge of *Arg*_38_ of *c* subunits, whereas on the cytoplasm side of IMM, a lyso‐phosphatidylserine (L‐PS) is bound to *Lys*_71_ of *e* subunit by ionic interaction [4]. The anionic phospholipids used, depending on the IMM leaflet, could envisage the hypothesis of lipid‐phase‐continuity H^+^ transfer for aerobic ATP synthesis [5]. The positive charge residue of the *Arg*_38_ side chain of each *c* subunit by facing the cavity of the *c*‐ring coordinates the negatively charged PS. The *Arg*_38_ residues of *c* subunits are placed at the end of the C‐terminal helix and form, together with *Asn*_39_ residues, the positive collar of the *c*‐loop at the boundary of the PS polar heads. The PS, inserted in the hole of the *c*‐ring with the two acyl chains, fills all the space. Moreover, the tight fit of the double‐chained matrix‐side lipid into the *c*‐ring establishes also hydrophobic interaction with a glycine zipper (G_20_xG_22_xG_24_xG_26_) and this suggests that the PS rotate with the *c*‐ring. The *Gly*_26_ → *Glu*_26_ mutation within the glycine‐rich region of *c* subunits is responsible for the mitochondrial permeability transition pore (mPTP)‐mediated hypoxia/reoxygenation cell death in cardiomyocytes [6] by missing the interactions of the acyl chains with the *c* subunits. Indeed, by the addition of negatively charged residue in the helix structure, it could favour the instability of phosphatidylserine in the *c*‐ring and its expulsion counted in the possible mechanism of the mPTP phenomenon [7].

## 2 THE PORE FORMS FROM THE *c*‐RING

**FIGURE 2:**
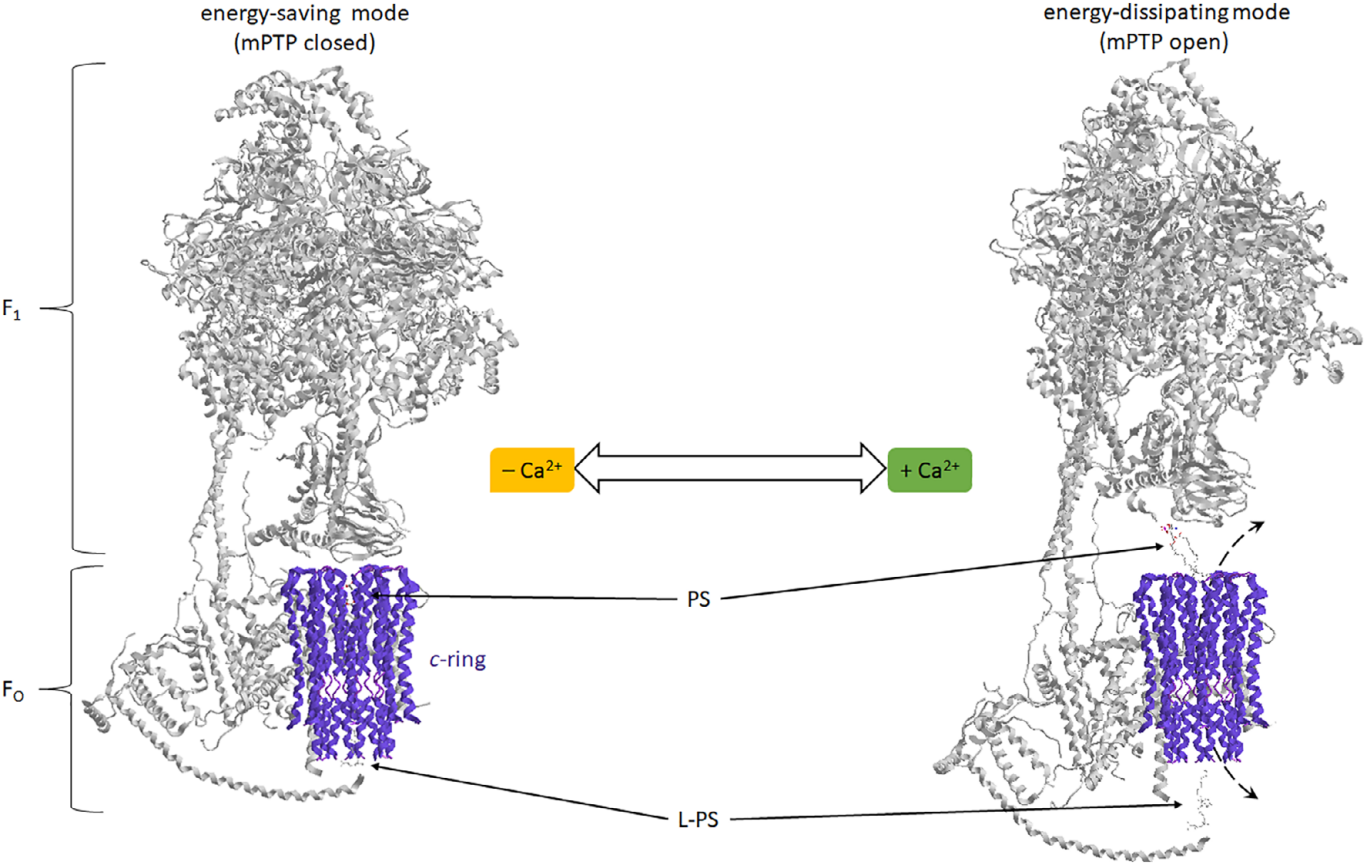
The dual‐mode of the F_1_F_O_‐ATPase. The structure of the mammalian mitochondrial F_1_F_O_‐ATPase (from modified PDB ID code: 6TT7) in the absence of Ca^2+^ cation (on the left). The suggested model of the PTP opening with the F_1_F_O_‐ATPase activated by Ca^2+^ (on the right). The enzyme forms the pore in the *c*‐ring when the lipid plugs are pushed/pulled away by (ir)reversible conformational changes. Phosphatidylserine (PS) and lyso‐phosphatidylserine (L‐PS) as a ball‐and‐stick model are indicated by arrows

Apart from being the main producer of ATP in mitochondria, the F_1_F_O_‐ATPase has other crucial roles in energy‐related homeostases, such as assisting mitochondrial *cristae* curvature and the cell death regulation via the mPTP [8]. Switching the energy transduction system from the energy‐saving to the energy‐dissipating mode is the newly discovered feature of the F_1_F_O_‐ATPase [9, 10]. The *c* subunits, which constitute the *c*‐ring, are considered the main components of the mPTP [4, 11, 12]. According to the new “bent‐pull” model [13], the Ca^2+^‐activated F_1_F_O_‐ATP(hydrol)ase activity generates a force transmitted from the F_1_ catalytic subunit to the membrane‐embedded subunit of the F_O_ domain. On the cytoplasm side of IMM, the lipid plug of L‐PS is pulled out of the *c*‐ring by the movement of the *e* subunit. On the other side of the *c*‐ring cavity, the PS coordinated with the *c* subunit fills the hole and blocks the pore. The water molecules coming into the *c*‐ring exert a thrust force on the PS [4]. The conformational change of the structure with the enlargement of the *c*‐ring could loosen the lipid interaction with the *c* subunits. The consequent modification of the *c*‐ring can also alter the interaction with the foot of the central stalk by favouring the detachment of the F_1_ from the F_O_ and the PS is pushed out creating a pore through the *c*‐ring [4, 10] (Figure 2).

## 3 THE HYPOTHESIZED EFFECT OF CyPD ON THE (UN)FOLDING OF THE *c* SUBUNIT

**FIGURE 3:**
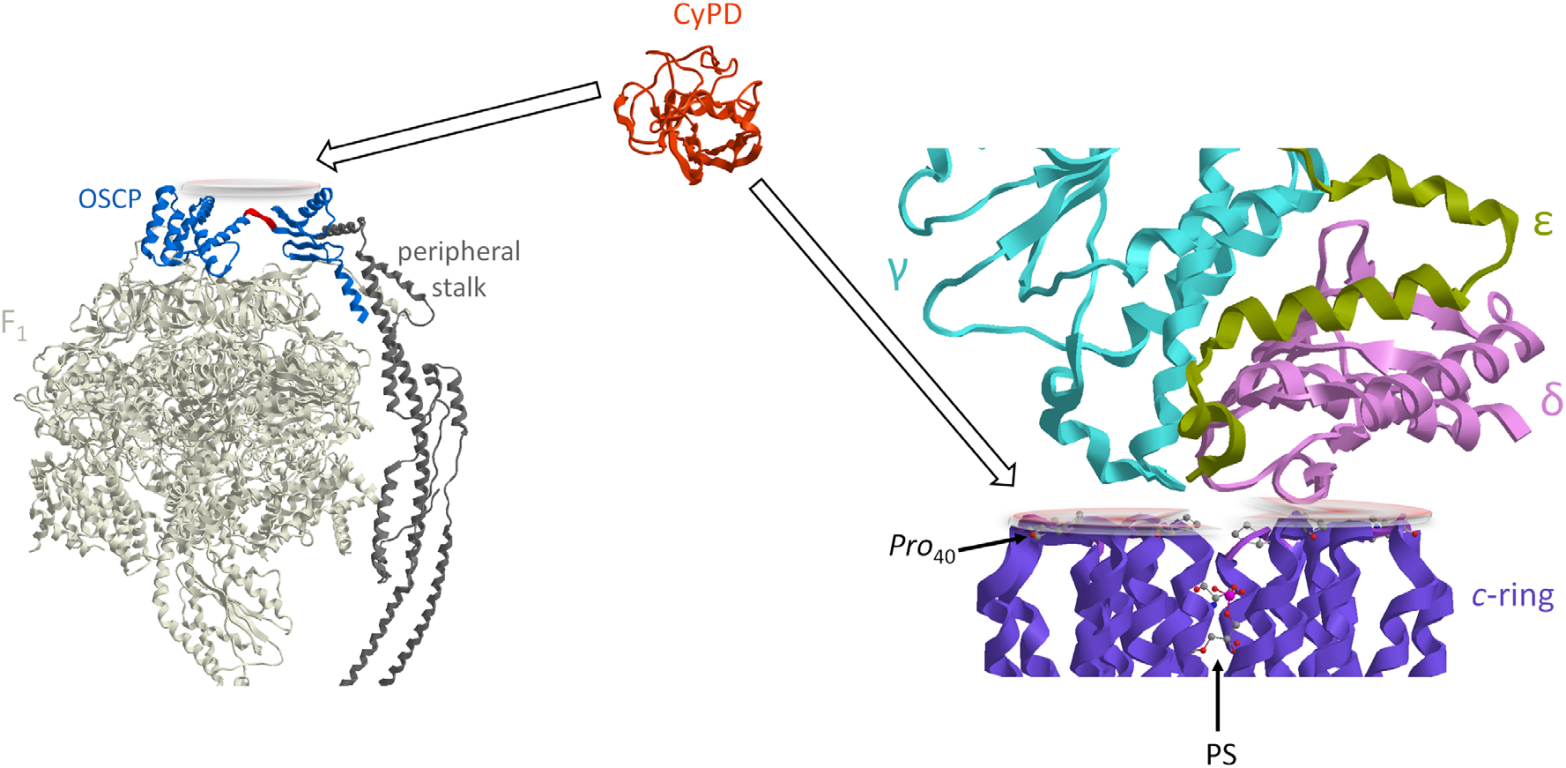
The CyPD binding sites of F_1_F_O_‐ATPase. On the left the hydrophilic portion of F_1_F_O_‐ATPase is obtained from modified PDB ID code: 6TT7. The interdomain hinge of the OSCP subunit is coloured red. On the right the rotor (the foot of central stalk: γ, δ, and ε subunit) and the *c*‐ring obtained from modified PDB ID code: 6TT7. The *Pro*_40_ and the phosphatidylserine (PS) are illustrated as a ball‐and‐stick model. The discs highlight the sites of CYPD (PDB ID code: 3QYU) interactions

Cyclophilin D (CyPD) is a mitochondrial chaperone protein identified as a peptidyl‐prolyl, *cis*‐*trans* isomerase (PPIase), which might be involved in mitochondrial protein folding, but there are no results on the presence of this activity [14]. Moreover, CyPD is a modulator of the mPTP formation and opening [15]. CyPD can bind the F_1_F_O_‐ATPase and has been suggested that the OSCP subunit of the peripheral stalk of the F_1_F_O_‐ATPase is its direct interactor in the enzyme complex [16]. The interdomain hinge of the OSCP subunit facilitates flexible coupling of the rotation to conformational changes of the catalytic subunits and makes this subunit an apposite point for the regulation of ATP synthesis [17]. The hinge flexibility of the N‐terminal OSCP domain linked to the F_1_ sector relative to the C‐terminal OSCP domain joined to the peripheral stalk is blocked by CyPD binding (Figure 3). This molecular event reduces the elastic movement of F_1_ with respect to the rotor permitting the signal transmission to the membrane‐embedded F_O_ sector where the pore opens [18, 19].

On the *c*‐loop that connects the two hairpin α‐helix of the *c* subunit is present the unique proline residue (*Pro*_40_) conserved between the F‐ and V‐type ATPase of different species, which could be a biological target of CyPD in the hydrophilic space placed between the *c*‐ring and the foot of the central stalk (Figure 3). Indeed, in cells overexpressing the *c* subunit, the mPTP is inhibited by cyclosporin A (CsA), the inhibitor of CyPD [12]. The CsA can inhibit the mPTP opening at an early stage but not at later ones. The *c* subunits could also form an ion channel by assembling into oligomers in a β‐sheet conformation with a similar mechanism to some other amyloidogenic peptides that form a β‐sheet oligomeric pore [20]. Recently, it has been suggested that CyPD‐*c* subunit interaction helps the formation of higher‐order oligomers, but is not required for pore activity by highlighting the folding activity in the mPTP formation [21].

**FIGURE 4:**
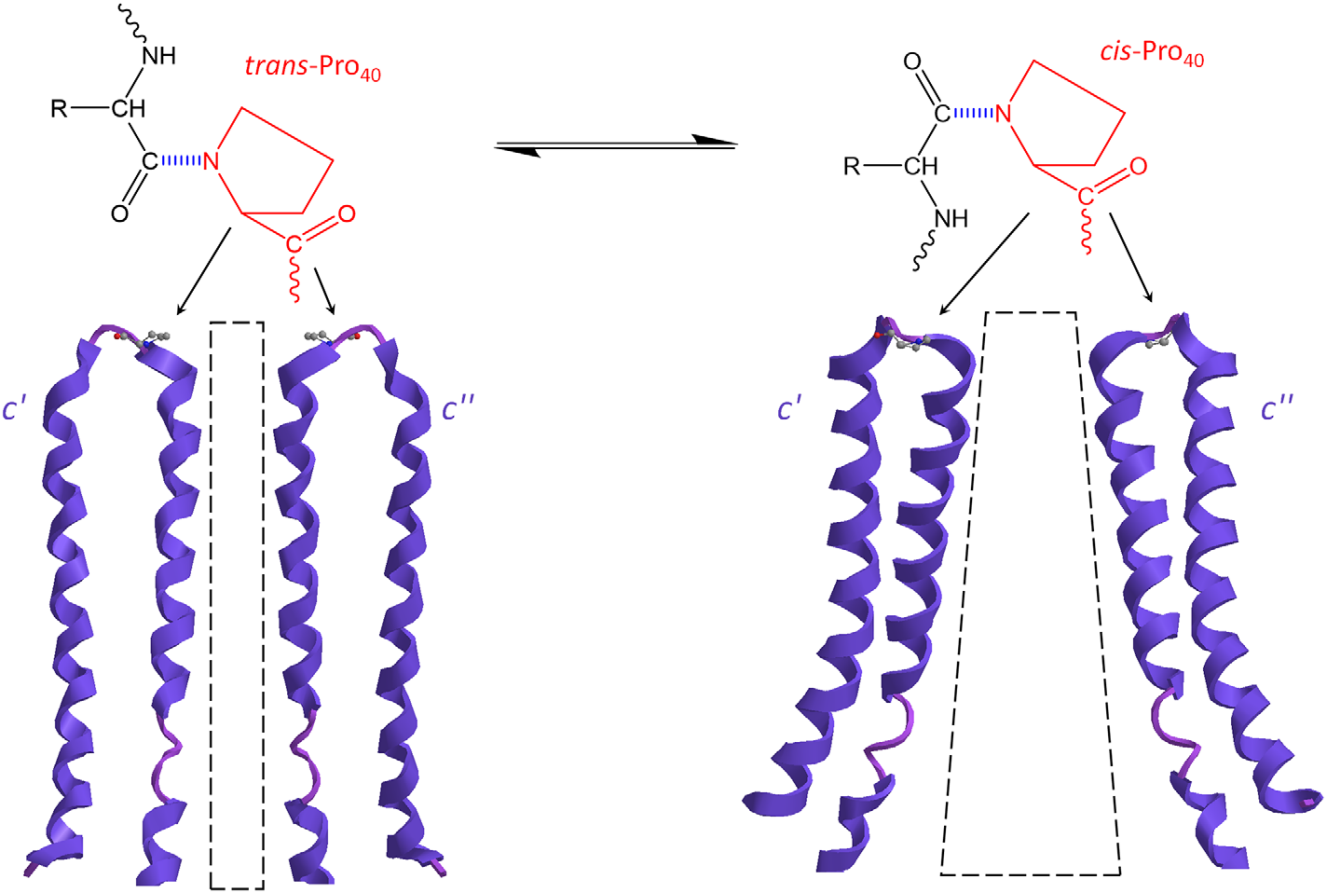
Proline isomers of *c* subunits. The immino nitrogen of proline (red structure) is involved in the torsional peptide bond (dashed blu) of the reversible *trans* and *cis* isomers. Two opposite *c* subunits (*c*’ and *c*”) of the *c*‐ring with the *Pro*_40_ in *trans* conformation (on the left) and *cis* conformation (on the right) show the difference of the hole (dotted shapes)

The *cis*‐*trans* isomerization of the *Pro* might provide the switch that interconverts the pore open/closed states by pulling out the *c*‐ring lipid plug. The *Pro* is the only amino acid with *cis*‐ *trans*‐isomerization in the peptide bond involving its imino nitrogen (Figure 4). Peptide bonds between amino acid residues are preferentially in the *trans* configuration, whereas the *cis* configuration occurs at β turns involving the *Pro* isomer. However, the *Pro* forms *cis* peptide bonds at a frequency higher than any other naturally occurring amino acid. The switch from *trans* to *cis* is a biological structural mechanism exploited in the channel opening [22, 23, 24]. A *Pro* located at the apex of the loop between two transmembrane helices can act as a hinge through a *cis‐trans* isomerization of the protein backbone. The hypothesis is that the natural *trans* configuration of the *Pro*_40_ allows the closed state of the pore. Conversely, if the N‐terminal helix of the *c* subunit works as a rigid body converting the *Pro*_40_ to the *cis* conformation the *c*‐ring enlarges the hole (Figure 4). The environment hydrated by incoming water molecules pulls the lipid plug out and obtains the open structure of the mPTP. Therefore, the *trans–cis* isomerization at *Pro*_40_ could function as a hinge for the movement of α‐helix during gating and explain the reported results that the *c* subunit alone without other parts of F_1_F_O_‐ATPase is sufficient to induce the mPTP formation [12].

## 4 CONCLUSION

The mitochondrial protein folding could be affected through the CyPD, but it might also achieve a scaffolding function, as it binds to several proteins in the mitochondrial matrix and the IMM [14]. The rotamase activity of PPIases increases the proline isomerization by up to 260 fold with an intramolecular hydrogen bond to the prolyl amide nitrogen [25]. If the CyPD has conserved structural features that facilitate *cis‐trans* isomerization, it is a kinetically viable candidate for the gating switch. On balance, this configurational stereoisomerism at a crucial pivot point on the *c*‐loop reorients the transmembrane helices of *c*‐subunits forming the pore. The *cis‐trans* isomerization of this single *Pro*_40_ could provide the switch that interconverts the open and closed states of the mPTP in a CyPD‐dependent manner through the *c*‐ring.

For instance, experiments with modified *Pro* analogues, which favour either the *cis* or the *trans* conformations, can provide further evidence on the role of *Pro* isomerization in F_1_F_O_‐ATPase, as done for the 5HT3A receptor [26]. Moreover, the recent development of photoswitchable amino acids [27] could provide an alternative way of testing the role of *Pro* isomerization in channel opening at the *c*‐ring. Completing such experiments, molecular dynamics simulations could give further insight into the mechanism of *Pro* isomerization in the *c*‐ring, as done for the 5HT3A receptor [28].

## CONFLICT OF INTERESTS

None.

### PEER REVIEW

The peer review history for this article is available at https://publons.com/publon/10.1002/prot.26383.

## Data Availability

Data sharing is not applicable to this article as no new data were created or analyzed in this study.
